# Characterization of the complete chloroplast genome sequence of *Daphne retusa* Hemsl. (Thymelaeaceae), a rare alpine plant species in northwestern China

**DOI:** 10.1080/23802359.2021.1944371

**Published:** 2021-06-28

**Authors:** Fang Yan, Chun-Yun Zhang, Qin-Li Wang, Jian-Dong Wang, Hai-Ping Wang, Tao Xu, En-Jun Wang, Liang-Yu Hou

**Affiliations:** a Key Laboratory of Hexi Corridor Resources Utilization of Gansu, Hexi University, Zhangye, China; b Taikang Pharmaceutical Limited Liability Company of Gansu Province, Wuwei, China; c Gansu Engineering Research Center for Resource Utilization of SMS, Hexi University, Zhangye, China; d Applied Technology Research Institute on original plants for Zushima of Hexi University, Zhangye, China

**Keywords:** *Daphne retusa*, Thymelaeaceae, complete chloroplast genome, phylogenetic relationship

## Abstract

*Daphne retusa* Hemsl. (Thymelaeaceae) is an evergreen shrub plant. First, we characterized the complete nucleotide sequence of chloroplast (cp) genome of *D. retusa*. The total length of cp genome was found to be 170,553 bp, including a large single copy (LSC) region of 84,886 bp, a small single copy (SSC) region of 2,437 bp, and a pair of inverted repeats (IRs) of 41,617 bp. The cp genome of *Daphne retusa* Hemsl. contains 134 genes, including 90 protein-coding genes (75 PCG species), 37 transfer RNA genes (29 tRNA species), and 6 rRNA genes (3 RNA species). A total of 13 genes (*trnK-UUU*, *trnS-CGA*, *atpF, rpoC1*, *trnL-UAA*, *trnC-ACA*, *petD*, *rpl16*, *rpl2*, *ndhB*, *trnE-UUC*, *ndhA*, and *trnA-UGC*) contain a single intron, and one gene (*ycf3*) contains two introns. The GC content in whole cp genome, LSC region, SSC region, and IR region was 36.75%, 34.83%, 28.19%, and 38.96% respectively, like other Thymelaeaceae plants. Phylogenetic analysis suggested that *D. retusa* has a close relationship with congeneric *Daphne tangutica*.

*Daphne retusa* Hemsl. (Thymelaeaceae) is a slow-growing evergreen shrub with a maximum height of 1 m, and it has flexible, off-white stems. This plant often grows on virgin land on hillsides at elevations of 2500–3000 m in the mountains in northwestern China. The dried stem and root bark of *D. retusa* are known as ‘ZuShima’ which is a common traditional Chinese medicine (Jamila et al. 2018). However, the wild sources of this plant have been nearly used up due to over-exploitation (Geng et al. 2016). In this study, we report the chloroplast genome sequence of *D. retusa*, and it would be essential to the conservation of this endangered species. The annotated genome sequence has been submitted to GenBank under accession number MW245832.

Fresh leaves of *D. retusa* were collected from Longnan Mountains (33°38′23″N, 104°50′30″E, 2910 m) in Gansu, China. The total DNA was extracted using the modified CTAB method (Allen et al. 2006) and used for the high-throughput Illumina HiSeq sequencing by Biomarker Technologies, Inc. (Beijing, China). A voucher specimen (ayrx_a) is deposited at the Building B2 of Kechuang 6 Road, Yard 88, Beijing Economic-Technological Development Area (E-mail: lingfeng_zeng@163.com). In total, 47,102,044 raw PE150 reads were obtained and quality-trimmed using the software in the NGSQC Toolkit v2.3.3 (Patel and Jain 2012) with 2% allowed ambiguous bases. The Geneious Prime (Kearse et al. 2012) was used to assemble the complete chloroplast genome. SPAdes v3.11.1 (http://cab.Spbu.Ru/software/SPAdes) was employed to obtain the optimal assembly results using multiple Kmer parameters (107, 117, 127), with the closely related species *Daphne tangutica* (GenBank: MK455800) serving as the reference. The assembled genome sequence was annotated using CPGAVAS2 (Biomatters Ltd., Auckland, New Zealand) by comparing it to the cpDNA of *Daphne tangutica* (GenBank: MK455800). Subsequently, a maximum likelihood (ML) tree with 1000 bootstrap replicates was inferred by MEGA7 with default parameters (Kumar et al. 2016). The iTOL (https://itol.embl.de/) was used for tree visualization.

The total length of nucleotide sequence of plastid genome is 170,553 bp, including a large single copy (LSC) region of 84,886 bp, a small single copy (SSC) region of 2,437 bp, and a pair of inverted repeats (IRs) of 41,617 bp (Figure 1). The cp genome of *D. retusa* contains 134 genes, including 90 protein-coding genes (75 PCG species), 43 transfer RNA genes (37 tRNA species), and 6 rRNA genes (4 RNA species). A total of 13 genes (*trnK-UUU*, *trnS-CGA*, *atpF*, *rpoC1*, *trnL-UAA*, *trnC-ACA*, *petD*, *rpl16*, *rpl2*, *ndhB*, *trnE-UUC*, *ndhA*, and *trnA-UGC*) contain a single intron, and one gene (*ycf3*) contains two introns. The GC content in whole cp genome, LSC region, SSC region, and IR region was 36.75%, 34.83%, 28.19%, and 38.96%, respectively, like other Thymelaeaceae plants.

**Figure 1.**
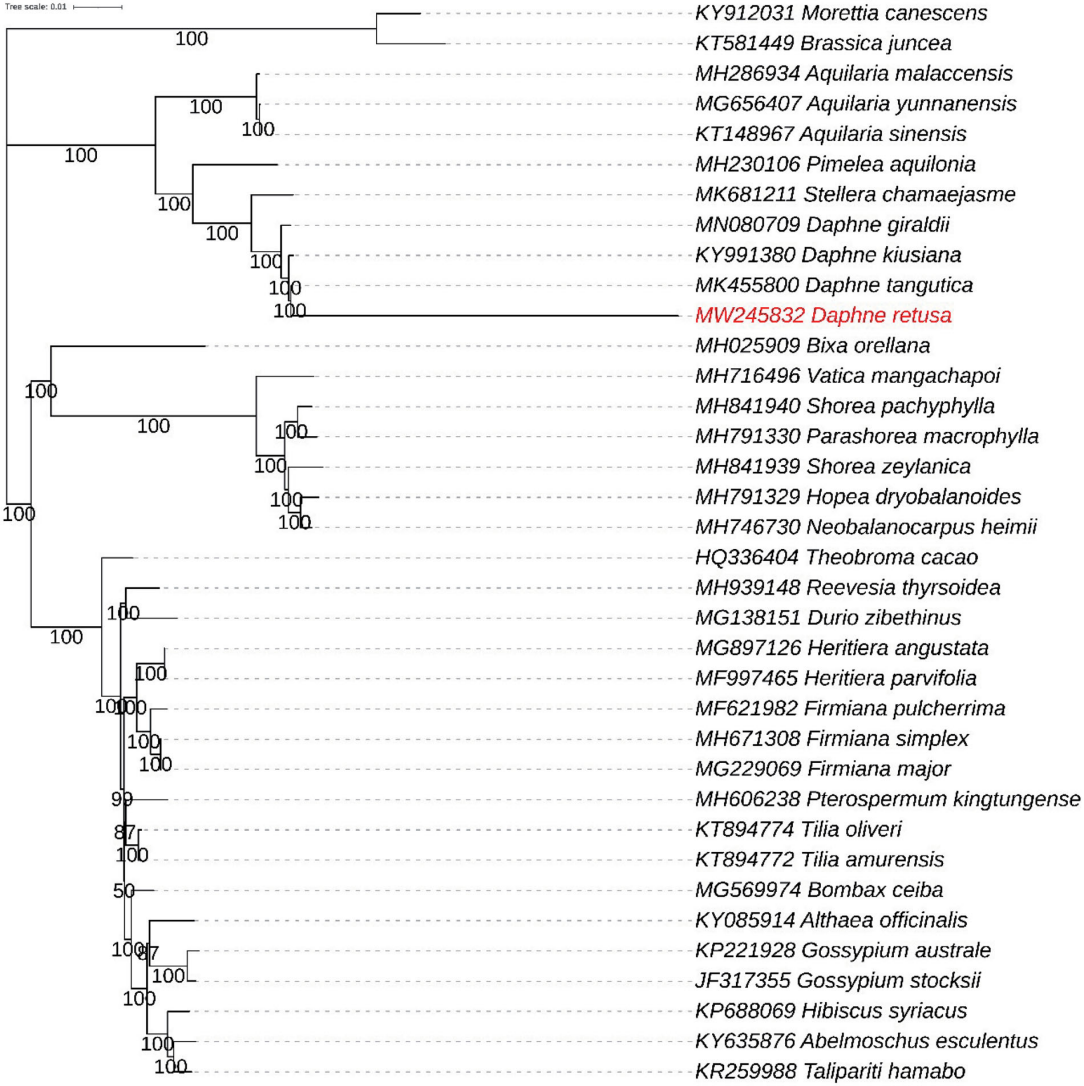
Maximum likelihood (ML) tree of *D. retusa* and its related relatives based on the complete chloroplast genome sequences.

To investigate the phylogenetic position of *D. retusa*, an alignment of Thymelaeaceae chloroplast genome sequences was processed by MAFFT (Katoh and Standley 2013), and a neighbor-joining (NJ) phylogenetic tree (Figure 1) was completed with MAFFT (Katoh and Standley 2013). The results showed close phylogenetic relationships among species in genus *Daphne*, especially among species *Daphne kiusiana*, *Daphne tangutica*, and *Daphne Giraldii*. The relationships within *Daphne* can be well distinguished using whole chloroplast genome data. *D. retusa* has a close relationship with congeneric *Daphne tangutica*. The complete chloroplast genome sequence of *D. retusa* facilitates the phylogenetic studies of Thymelaeaceae.

## Data Availability

The genome sequence data that support the findings of this study are freely available in GenBank at (https://www.ncbi.nlm.nih.gov/) under accession number MW245832. The associated BioProject, SRA, and Bio-Sample numbers are PRJNA693110, SRX9895515, and SAMN17377581, respectively.
